# Brief Remarks on the Treatment of Fistula Lacrymalis by Dilatation

**Published:** 1843-07-08

**Authors:** Isaac Parrish

**Affiliations:** The Surgeons to Wills’ Hospital


### THE MEDICAL EXAMINER,

### And Retrospect of the Medical Sciences.

Vol. VI.] PHILADELPHIA, SATURDAY, JULY 8, 1843. [No. 13.

### BRIEF REMARKS ON THE TREATMENT OF FISTULA LACRYMALIS BY DILATATION.

BY ISAAC PARRISH, M. D.

One of the Surgeons to Wills' Hospital.

In several cases of fistula lacrymalis, which have recently fallen under my care, a plan of treatment has been pursued, differing in some respects from that generally recommended; and sufficiently satisfactory in its results to warrant a public notice.

The principle of treatment is not new; it is precisely that which is recognized by the best authorities, in the management of strictures of the urethra, by the gradual and steady operation of bougies of various sizes. The two affections are indeed very similar; in both a mucous canal is obstructed by the thickening of its internal surface, under the influence of chronic inflammation; whereby the fluids destined to pass through it, are impeded in their course.

In the case of the nasal duct, when the obstruction is complete and permanent, the lacrymal sac becomes inflamed, or distended to such a degree, as either to burst spontaneously, or to require an opening—thus forming a fistulous opening over the sac.

The obvious indication under these circumstances, is to re-establish the passage to the nose, and thus to relieve the patient from the unpleasant consequences of a constant accumulation of fluids within the lacrymal sac.

In mild cases of this description, injections into the sac and duct by Anel's syringe, the passage of probes, and the adoption of a course of treatment to reduce inflammation, has been found sufficient to effect a cure. In the large proportion of cases, however, a more energetic practice becomes necessary. The course generally recommended, and practised, is to enlarge the fistulous opening, and insert into the duct the nail headed style of Ware, which is worn by the patient, and is said to secure the passage of the tears to the nose.

Never having used this instrument, I cannot speak from experience of its merits, having always been able to effect the desired object by less objectionable means.

The plan pursued has been, first to reduce any inflammation which may exist around the fistulous sore, by means of emollient poultices, and then introduce a small piece of fine wax bougie, having an acute point, down to the strictured part, (or as far as it can be inserted,) and by turning over its blunt end to secure it there by means of an adhesive strip drawn firmly over it. This may be removed in a day or two, and after washing out the parts, may be reintroduced, or one of a larger size inserted, if it can be borne. At the commencement of the treatment little more will be accomplished than to dilate the fistulous orifice, and to induce a more healthy action in the mucous surface of the sac and duct, but by perseverance in the plan, an advance will be made from day to day upon the strictured surface; until, finally, a bougie can be passed through the whole extent of the canal. This being accomplished, the dilatation should be continued, until the bougie will pass freely, and until all hardness and inflammation has disappeared from around the fistulous orifice—when it may be permitted to heal up, which it will usually do under simple dressings.

By this process the canal is gradually dilated, and its mucous surface restored to a healthy condition, without doing violence to the parts, while the duct is placed in a condition to resume its natural functions, without the necessity of permanent dilatation. If, however, there should be a disposition to relapse, after the closure of the orifice, it may be counteracted by the use of stimulating ointments to the inner surface of the lids, by astringent washes, or by such other means as circumstances may indicate, preventing, if possible, the reopening of the fistulous sore.

Beer, the German oculist, has recommended the use of catgut to force the obstructed duct. A piece of catgut of the ordinary length and size of a small fiddlestring is passed through the duct into the nose, one extremity is brought out and fixed near the ala nasi by sticking plaster; and the other secured to the brow by a turn or two of bandage. Every day a fresh portion of catgut is drawn through, until several sizes have been made to pass, when the cure is considered complete, and the fistula is allowed to heal.

A serious objection to this method is found in the difficulty of effecting the passage of the duct, at one operation, by any instrument, however fine, and should the operator succeed, the force required would be apt to tear the mucous surface and excite a degree of inflammation, very unfavourable to the ultimate restoration of the parts; whereas, by the gradual process here recommended, the difficulty is slowly overcome, while, at the same time, the diseased mucous surface is restored to healthy action, and the integrity of the canal is preserved.

Indeed, one of the great advantages of the bougie made of waxed linen, is its bland and salutary action on a surface which has been thickened by chronic inflammation. This is evinced by the increase of mucous discharge which follows its introduction, and in the gradual return of the membrane to its normal state. The same remark applies to strictures of the urethra, and hence it was that the late Dr. Physick and others preferred the bougie of waxed linen, for the treatment of stricture, to those of more modern invention.

The instrument is made by dipping a piece of fine linen into white wax in a melting state, and suddenly withdrawing it. It is then allowed to cool, and cut into portions which, when tightly rolled, form a bougie, of any size which may be required.

Another advantage possessed by this instrument, is, that it may be cut or bent with great ease, and be made smaller by unwrapping, without the necessity of having a great number of sizes already prepared.

It may be mentioned, also, that this form of bougie is very useful in the treatment of fistulous sores or sinuses, where it is important to excite a moderate degree of inflammation.

The introduction of a bougie of suitable size into a sinus, and its retention there by appropriate dressings will often be found more effectual in promoting healthy action, than the use of stimulating injections, or other more violent means.

